# From structural complexity to circular applications: a comparative review of the enzymatic degradation of polydopamine, tannins, lignins, and melanins

**DOI:** 10.1039/d5ra08182c

**Published:** 2026-05-11

**Authors:** Ruchika Atri, Sascha Putzke, Frank Simon, Cordelia Zimmerer

**Affiliations:** a Leibniz-Institut für Polymerforschung Dresden e.V. Hohe Straße 6 D-01069 Dresden Germany zimmerer@ipfdd.de

## Abstract

Polydopamine, tannins, lignins, and melanins are diverse natural and bio-inspired polyphenolic materials known for their structural complexity, functional versatility, and growing technological importance. Their widespread presence in biological systems and emerging applications in coatings, packaging, biomedicine, and sustainable materials call for a deeper understanding of their biodegradation. This review presents a comparative analysis of their enzymatic breakdown, highlighting the roles of oxidoreductases, including laccases, peroxidases, and tyrosinases, in facilitating polymer depolymerization, structural modifications, and downstream valorization. Unique structural features such as catechol units in polydopamine, galloyl groups in tannins, phenylpropanoid backbones in lignins, and indole-quinone frameworks in melanins dictate their degradation rates and enzyme accessibility, presenting specific challenges and opportunities. Beyond biodegradation, this review situates these processes within the framework of potential recycling-by-design and a circular economy, demonstrating how controlled enzymatic conversion can produce high-value intermediates for green chemistry, biomaterials, energy recovery, and environmental remediation. By comparing these biopolymers side by side, we identify common principles, challenges, and technological opportunities to transform enzymatic degradation into a key driver of circular bioeconomy strategies. To ensure relevance and accuracy, only recent research published since 2015 has been included, focusing on the latest advances in enzymatic degradation and sustainable applications. This synthesis fills a critical knowledge gap, serving as a valuable resource for researchers, industries, and policymakers seeking to develop innovative, sustainable materials that align with the principles of green chemistry and environmental responsibility.

## Abstract 2

Polydopamine, tannins, lignins, and melanins are diverse natural and bio-inspired polyphenolic materials known for their structural complexity, functional versatility, and growing technological importance.

## Graphical abstract

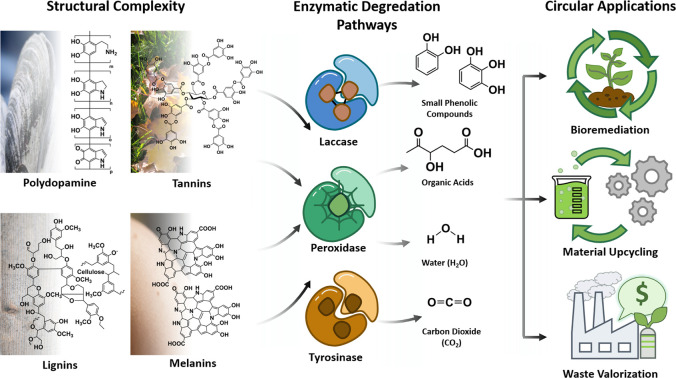

## 1 Introduction

The accumulation of synthetic polymers has led to significant environmental challenges, including persistent plastic pollution and inefficient waste management systems. Conventional polymers derived from petrochemical sources are somewhat challenging to biodegrade, raising long-term ecological and health concerns.^1^ The emergence of sustainable materials and green chemistry has shifted the focus to natural biopolymers produced from renewable sources.^2,3^

Among promising alternatives, biopolymers such as polydopamine (PDA), tannins, lignins, and melanin have attracted considerable attention for their unique chemical structures and multifunctional properties. These naturally derived polymers provide environmentally friendly alternatives to petroleum-based materials, exhibiting advantageous characteristics such as antioxidation, strong adhesion, and structural integrity. These materials belong to the broader class of bio-derived functional polymers, characterized by high aromaticity, redox activity, metal-chelating ability, and strong interfacial adhesion. Their renewable origin and multifunctionality enable them to replace petroleum-based polymers in several application fields. Polydopamine is increasingly used in surface coatings, biomedical interfaces, and adhesive technologies.^4^ Tannins serve as natural precursors for wood adhesives, flame-retardant systems, and antioxidant additives.^5,6^ Lignin, a hydroxylated aromatic biopolymer abundant in plant cell walls, has gained attention as a sustainable alternative to petroleum-based additives due to its biodegradability and favorable rheological, mechanical, chemical, and thermal properties. In addition to its established use in bioplastics, composites, and polymer formulations, lignin has shown strong potential in oilfield applications. Its incorporation into drilling fluids, fracturing fluids, enhanced oil recovery (EOR) systems, and oilfield wastewater treatment systems provides an environmentally friendly alternative to conventional petrochemical-derived materials.^7,8^ Melanin is applied in UV-protective coatings, bioelectronics, and photoprotective materials.^9^ Recent studies highlight their potential to reduce dependence on petrochemical polymers while enabling sustainable material design.

The capacity to enzymatically degrade these biopolymers is a key advantage, distinguishing them from synthetic plastics that persist in ecosystems for centuries.

Enzymatic degradation, mediated by oxidative enzymes such as laccases, peroxidases, and tyrosinases, plays a crucial role in regulating the biodegradability and recycling potential of these biopolymers.^10^ The enzymatic processes in their polymerization and degradation are fascinating, as they influence material formation and prospects for recycling or repurposing. Enzymes, recognized as eco-friendly and non-toxic catalysts, offer a sustainable alternative to traditional chemical catalysts, which often involve toxic metals that require complete removal, especially in biomedical applications.^11^

The use of enzymatic processes in polymerization and depolymerization presents several advantages. Unlike conventional chemical methods, enzymatic synthesis does not necessitate the stringent removal of moisture and oxygen, making it particularly beneficial for preserving thermally or chemically sensitive functional groups during polymerization.^12^ Additionally, immobilized enzymes offer enhanced stability against environmental fluctuations and can be quickly recovered and reused, making them more advantageous than their free counterparts. Beyond reusability, proper immobilization techniques protect enzymes from harsh conditions, such as extreme temperatures and pH levels, while enhancing key enzymatic properties. These include increased purity, stability (with potential for reactivation), activity, specificity, selectivity, and reduced inhibition. Consequently, enzyme immobilization enhances industrial applicability and cost efficiency, making it a valuable strategy for sustainable biocatalytic processes.^13–15^

Beyond polymer synthesis, enzymatic recycling strategies enable the degradation of biopolymers into recoverable monomers, promoting a circular economy by facilitating the reuse of materials.^16,17^ This approach contributes to waste reduction and aligns with the principles of sustainable material innovation and green chemistry.^18^

Despite these advantages, significant knowledge gaps remain regarding the enzymatic degradation pathways of these biopolymers, their stability under varying environmental conditions, and the optimization of these processes for large-scale applications. While individual studies have explored aspects of their biodegradability and applications, a comprehensive review that consolidates recent findings from 2015 onward is lacking.

This review addresses this gap by providing an in-depth analysis of the biological origins, chemical compositions, enzymatic degradation mechanisms, and industrial applications of polydopamine, tannins, lignins, and melanin. We emphasize a comparative perspective, discussing how these biopolymers differ and overlap in terms of enzymatic degradation pathways and applications across diverse fields, thereby clarifying their potential roles in advancing sustainable material science, reducing environmental impact, and enabling circular applications. Furthermore, by situating these comparisons within the framework of green chemistry principles-including the use of renewable feed stocks, eco-friendly enzymatic catalysts, waste reduction, and recyclability-this review highlights how PDA, tannins, lignins, and melanin contribute to the development of safer, cleaner, and more sustainable material technologies.

This review is a valuable resource for researchers, industry professionals, and policymakers seeking to integrate biodegradable, bio-based materials into various sectors.

Table 1 summarizes those tannins degrade fastest due to simple structures; PDA shows moderate enzymatic degradability and stability; lignins are highly resistant needing special enzymes; melanins are extremely resistant, offering long-term stability. The degradability order—tannins > PDA > lignins > melanins—reflects how complexity and groups affect enzyme access and circular use.

**Table 1:** Comparative overview of polydopamine, lignins, tannins, and melanins

Biopolymer or macromolecule	Origin and structure	Application	Stability/biodegradability	Key enzymes in degradation	Degradation pathway	References
Polydopamine (PDA)	Synthetic analogue of melanin; catechol- and amine-rich polymer formed *via* oxidative self-polymerization of dopamine	Surface coatings, adhesives, photothermal therapy, drug delivery, biosensors	Moderate stability; degradable by oxidative enzymes but stable under physiological conditions	Laccases, peroxidases, tyrosinases	Oxidative cleavage of catechol and amine groups → smaller catecholamine derivatives	19–37
Tannins	Plant-derived polyphenols; hydrolyzable (esters of gallic/ellagic acid) and condensed tannins (proanthocyanidins)	Natural antioxidants, adhesives, foams, tanning agents, pharmaceuticals, food additives	Readily biodegradable; hydrolyzable tannins degrade faster than condensed tannins	Tannase, laccases, peroxidases	Hydrolyzable tannins → gallic/ellagic acid; condensed tannins → flavonoid monomers	38–73
Lignins	Natural aromatic polymer in plant cell walls; irregular, cross-linked structure with guaiacyl, syringyl, and *p*-hydroxyphenyl units	Biofuels, resins, carbon fibers, bioplastics, sustainable composites, biorefinery feedstock	Highly recalcitrant; slow and incomplete biodegradation	Lignin peroxidase, manganese peroxidase, laccases (from white-rot fungi)	Radical-mediated oxidative cleavage of C–C and C–O bonds; depolymerization into phenolic compounds	74–149
Melanins	Biological pigments; amorphous indolic/phenolic-based macromolecules in fungi, animals, bacteria, plants	UV protection, radioprotection, biomedical coatings, electronic/optical materials, antioxidants	Extremely stable against chemical, thermal, and photodegradation; very slow biodegradation	Peroxidases, tyrosinases, microbial oxidases	Oxidative cleavage of indolic/phenolic units; typically incomplete and very slow	150–212

## 2 Polydopamine (PDA): inspired by the mussel's adhesive

PDA is a bioinspired polymer derived from the oxidative polymerization of dopamine (DA). It is inspired by the nature's adhesive foot proteins of mussels of the *Mytilus* spp. (such as *Mytilus edulis*) because of its unique surface coating capability and abundant active sites, which are known for their strong adhesion, biocompatibility, and multifunctional properties.^19,20^ Since its discovery in 2007, PDA has gained significant attention across various scientific fields, including biomedicine, materials science, and environmental engineering.^21^

**Fig. 1:**
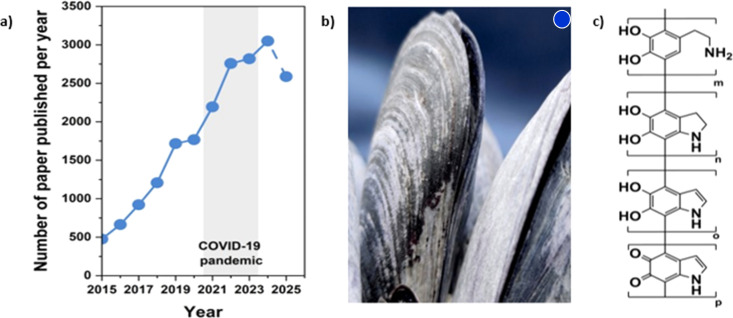
(a) Graph illustrating the total number of scientific papers published on PDA, their structures, biodegradation, and their use from 2015 to 2025. The dot for 2025 represents papers published through December (source: *Web of Science*, 2025-12-31). (b) Image showing the natural biological inspiration for PDA, derived from mussel adhesive chemistry. (c) Representative structural motifs associated with the polymeric framework of polydopamine.

Fig. 1 highlights the accelerating expansion of research on polydopamine (PDA) over the past decade. As shown in panel (a), publications related to PDA synthesis, structure, biodegradation behavior, and emerging applications have increased continuously from 2015 to 2025, with the 2025 data point reflecting all entries indexed up to 31 December 2025. This sustained rise underscores the broadening interest in PDA as a functional material for coatings, catalysis, biomedicine, environmental remediation, and sustainable materials design. Panel (b) depicts the marine mussel, the natural origin of the catechol-based adhesion chemistry that inspired PDA and continues to motivate investigations into wet-surface interactions and bioinspired interfacial engineering. Panel (c) illustrates representative PDA structural motifs, which highlight the heterogeneous and partially unresolved nature of the polymer formed through catechol oxidation, intramolecular cyclization, and mixed covalent–noncovalent crosslinking. Together, the three panels emphasise the synergy among biological inspiration, complex molecular architecture, and the expanding technological relevance that has driven the rapid growth of PDA-focused research.

Structurally, PDA exhibits chemical functionalities similar to those of mussel adhesive proteins, particularly *Mytilus edulis* foot protein-5 (Mefp-5), which is rich in catechol (1,2-dihydroxybenzene) and nitrogen-containing groups.^22^

These functional groups are responsible for the firm adhesion of mussels to various surfaces, even under extreme marine conditions, due to the presence of catechol and amine-rich amino acids in their adhesive threads.^23,24^ PDA's chemical structure and resulting universal adhesion properties remain controversial, with proposed models suggesting a melanin-like composition composed of catechol, quinone, and indole units. The polymerization process involves oxidation and reduction steps, associated with intramolecular and intermolecular cross-linking, resulting in an amorphous, complex chemical structure.^25^ Advanced spectroscopic studies have provided insights into the morphology and molecular organization of PDA coatings, highlighting their π–π stacking interactions and hydrogen-bonding capabilities.^24^

Inspired by this natural adhesion mechanism, researchers have developed synthetic materials incorporating similar functional groups, with applications extending beyond adhesives to biomedicine, nanotechnology, and surface coatings.^26^ PDA is remarkable for its ability to spontaneously deposit onto virtually any surface, regardless of its chemical composition, making it a highly versatile material for surface functionalization.^27^

### 2.1 Enzymatic degradation

Enzymatic degradation of PDA represents a promising, eco-friendly strategy for modulating its stability across various applications. Laccases and peroxidases have demonstrated significant potential in catalyzing PDA breakdown, with implications in biomedical applications such as drug delivery and tissue engineering,^213^ environmental remediation,^214,215^ and material sciences.^216^

Oxidative enzymes, particularly laccases and peroxidases, are crucial in the enzymatic degradation of PDA. Laccase, a copper-dependent oxidase, facilitates the oxidation of catechol and dopamine derivatives, influencing both the formation and decomposition of PDA.^28,29^ Furthermore, copper-chelated PDA nanozymes exhibiting laccase-like activity have been explored for the oxidative degradation of organic dyes, highlighting their potential applicability in PDA degradation.^29^

Laccase enzymes, particularly from fungal and bacterial sources, have shown substantial promise in the degradation of PDA due to their ability to oxidize phenolic substrates. Recent work by Palaskar *et al.*^30^ demonstrated that laccase from *Klebsiella oxytoca* immobilized on sodium alginate efficiently degraded organic dyes, suggesting similar potential in PDA degradation. In addition to laccase, peroxidases such as lignin peroxidase (LiP) and manganese peroxidase (MnP) have been instrumental in degrading complex organic polymers, including PDA, as evidenced by studies on lignite degradation by mixed white-rot fungi.^31^

Moreover, enzyme-based approaches have expanded to include tyrosinase-mediated oxidation, where PDA-assisted hydrogels embedded with tyrosinase have been employed for phenol degradation in contaminated water.^32^ Recent advancements in PDA degradation also highlight the use of peroxidase-mimicking nanozymes, such as Co_3_O_4_/PDA composites, which exhibit robust catalytic activity under near-infrared light, effectively breaking down phenolic and polymeric pollutants.^33^ These studies underscore the potential of enzymatic and nanozyme-based strategies for PDA biodegradation, offering environmentally friendly alternatives for managing PDA-based materials.

Despite promising findings, several challenges remain. Enzyme stability under varying environmental conditions, the specificity of degradation pathways, and the scalability of enzyme-based degradation processes need further investigation. Recent studies have explored peroxidase-based enzyme immobilization techniques to enhance catalytic stability and reusability.^217^ Additionally, nanozyme-based strategies, which mimic enzymatic activity, offer potential for enhanced PDA degradation efficiency in industrial applications.^218^

Future research should focus on optimizing enzyme immobilization methods, engineering more efficient oxidative enzymes, and exploring synergies between enzymatic and photocatalytic degradation processes. Developing multi-functional enzyme-nanozyme hybrid systems could provide a scalable and sustainable approach to PDA degradation.

### 2.2 Applications of PDA

#### 2.2.1 Biomedical applications

PDA is a valuable biopolymer with diverse applications thanks to its advantageous properties. Its ability to scavenge free radicals, efficiently convert light to heat, and provide excellent biocompatibility and biodegradability, coupled with its inherent fluorescent and theranostic capabilities due to its versatile surface chemistry, make it a promising material for the biomedical field.^24,34,219^

Recent reviews have reinforced these insights, emphasizing PDA's multifunctional roles across drug delivery, tissue engineering, biosensing, antimicrobial therapy, and environmental applications.^220^ PDA has been extensively utilized as a carrier for drug delivery due to its high drug-loading capacity and stimuli-responsive release mechanisms. Recent studies highlight the role of PDA-based nanosystems in targeted drug delivery, particularly in cancer therapy.^221^ More recent work provides mechanistic clarity, showing that particle size, morphology, and surface charge strongly influence circulation behavior, tumor penetration, and drug-release efficiency.^222^ Additionally, PDA-coated MOFs and hollow mesoporous PDA systems now enable combined chemo-photodynamic-immunotherapy, offering stronger tumor inhibition and controlled-release profiles.^223^ It is also being explored in novel biohybrid drug carriers. For instance, a *Lactobacillus*-PDA system was developed for targeted drug delivery in the treatment of overactive bladder, demonstrating effective penetration into bladder tissues and improved therapeutic efficacy.^224^ PDA-functionalized titanium dioxide nanotube arrays have been proposed for sustained local drug delivery, ensuring long-term therapeutic effects in implant-related applications.^33^ Beyond oncology, PDA-based nanotechnologies have expanded into neurological therapeutics, where they can reduce oxidative stress, suppress neuroinflammation, and enhance BBB permeability, showing promise for the management of stroke, Parkinson's disease, spinal cord injury, and glioma.^225^

In tissue engineering, PDA is used for its ability to enhance biomaterial–cell interactions and promote cellular adhesion and proliferation. PDA's ability to bind various substrates has also been exploited to functionalize composite scaffolds for tissue engineering, thereby enhancing hydrophilicity and cellular integration.^226^ PDA coatings on scaffolds have improved biocompatibility and osteo-inductive properties, making them highly suitable for bone tissue engineering.^227^ Recent literature highlights PDA's emerging role in 3D-printed scaffolds, biosensing-active constructs, and multifunctional regenerative biomaterials, further strengthening its importance in regenerative medicine.^228^ In addition, PDA-modified polycaprolactone [poly(hexano-6-lactone)] scaffolds loaded with metal nanoparticles have demonstrated antibacterial and osteogenic capabilities, facilitating improved bone regeneration.^229^

In nerve tissue engineering, PDA-functionalized polyurethane/graphene oxide scaffolds are designed to regulate the inflammatory response and promote axonal regeneration, offering a promising approach for peripheral nerve repair.^224^ PDA has also been used to functionalize nanohydroxyapatite-coated exosomes, significantly enhancing their cytocompatibility and osteogenic potential.^229^

PDA has demonstrated excellent antibacterial properties, particularly when combined with metal nanoparticles. Silver-decorated PDA nanoparticles have been developed as photothermal platforms for bacterial eradication and accelerated wound healing,^230^ and PDA-based hydrogels have been explored for self-powered transdermal drug delivery, providing a non-invasive approach for controlled therapeutic administration.^231^ These findings align with recent reviews that position PDA nanoparticles as smart antimicrobial materials capable of disrupting biofilms, modulating ROS, and synergizing with metal ions or peptides.^232^

Recent advancements have explored PDA-based nanomedicines for their antiviral properties. A study developed a PDA-based system that exhibited efficient antiviral activity against SARS-CoV-2 (severe acute respiratory syndrome coronavirus 2).^233^ This system demonstrated outstanding therapeutic effects, including the improvement of pulmonary edema and protection against lung injury in a mouse model of COVID-19 (coronavirus disease 2019). Liu *et al.*^234^ developed a PDA-based lateral flow immunoassay (LFIA) for accurate, large-scale COVID-19 screening. Compared to conventional LFIA methods, their approach offers two key innovations: PDA's broader, stronger visible absorption enhances sensitivity over gold nanoparticles, and the three-line LFIA enables simultaneous detection of IgG and IgM (immunoglobulin G and immunoglobulin M).

This advancement supports effective outbreak control and self-detection. A novel nano-bait strategy utilizing exosome-sheathed PDA nanoparticles was introduced to inhibit SARS-CoV-2 infection. These nanoparticles, which display ACE2 (angiotensin-converting enzyme 2) receptors on their surface, effectively trap the virus and concurrently reduce inflammation by scavenging reactive oxygen species, offering a dual-action therapeutic approach.^235^

PDA's optical and electrical properties make it valuable material for biosensing and bioimaging applications. PDA enhances biosensing by simplifying detection, improving stability, and increasing sensitivity. It enables user-friendly sensors for early disease detection, environmental monitoring, and safety, offering superior accuracy for biological and chemical targets.^236,237^ PDA-functionalized nanoparticles have been used for nucleic acid-based biosensing, enabling highly sensitive detection of biomolecules.^238^ Moreover, PDA-coated fluorescent nano-diamonds have been proposed as versatile platforms for bioimaging and cancer diagnostics.^35^ These findings align with recent advancements that position PDA as a broadband optical indicator for colorimetric and fluorescence-based sensing, thanks to its tunable optical absorption and high signal stability.^239^ Its ability to serve as a platform for secondary functionalization has been exploited in implant coatings to enhance osseointegration and prevent bacterial infections.^224^

#### 2.2.2 Material science

PDA has significantly advanced surface engineering by enabling universal and facile surface modification of metals, polymers, glass, ceramics, and both hydrophilic and hydrophobic substrates.^240^ This broad substrate compatibility continues to make PDA an attractive platform for adhesion enhancement, corrosion protection, metallization, and energy-related materials. Recent research further reinforces PDA's versatility: for example, metal-PDA coordinated systems have been shown to modulate corrosion resistance, antioxidant retention, and antibacterial activity through metal-specific interaction pathways.^241^

Zimmerer *et al.*^242^ previously demonstrated that dopamine (DA) and PDA provide a promising interfacial design for environmentally friendly metallization of plastics, reducing energy consumption and eliminating harsh etching chemicals. These findings align with recent work showing that metal-ion-containing PDA films (*e.g.*, Fe^3+^-PDA, Cu^2+^-PDA) can markedly improve polymer metal adhesion by modifying interfacial chemistry and increasing pull-out energy.^243^ Such studies collectively demonstrate a broader trend: PDA is now viewed not merely as a passive adhesive layer but as a tunable coordination network whose metal-binding chemistry can be engineered to meet specific mechanical and processing requirements.

PDA coatings have been particularly effective in protecting metal substrates such as stainless steel, magnesium alloys, and carbon steel. Moreover, PDA-based coatings have been developed for corrosion inhibition in metal surfaces, offering an environmentally friendly alternative to traditional coatings.^244^ Li *et al.*^245^ explored the grafting of polyaniline (PANi) onto PDA-wrapped carbon nanotubes to enhance corrosion protection in epoxy coatings. This hybrid approach significantly improved the barrier properties and longevity of the protective layer. PDA has also been incorporated into polymeric coatings to enhance their anti-corrosion performance. Cheng *et al.*^244^ demonstrated that PDA-modified ultrathin hydroxyapatite nanosheets significantly improved the corrosion resistance of polymeric coatings. Similarly, Cui *et al.*^246^ explored bioinspired PDA nanosheets for reinforcing waterborne epoxy coatings, resulting in superior adhesion and corrosion resistance. Recent studies (2025) strengthen this by revealing that adjusting PDA ratios in epoxy systems significantly improves long-term corrosion resistance, hydrophobicity, and nanofiller dispersion, providing mechanistic insights into PDA-based anticorrosion design.^247^ Similarly, Zimmerer *et al.*^248^ coated commercial phase change material (PCM) microcapsules with an ultrathin self-polymerized PDA film, followed by nickel metallization. The deposited PDA layer was thinner than most reported in the literature, resulting in lower mass deposition and higher residual specific phase change enthalpy. Despite the PDA/nickel coating, the microcapsules maintained efficient heat storage and release performance.

Additionally, Chen *et al.*^249^ reported that PDA-decorated stannic oxide (SnO_2_) nanocontainers provide an effective corrosion barrier for stainless steel surfaces, highlighting PDA's versatility in metal protection.

PDA-derived materials have been explored for energy storage applications, *e.g.*, rechargeable batteries and supercapacitors, thereby enhancing electrochemical performance.^250^ Zhu *et al.*^251^ developed nitrogen and oxygen co-doped carbon nanosheets synthesized from PDA, significantly enhancing supercapacitor performance due to improved electron transport and surface reactivity. Similarly, PDA-functionalized porous carbon nanofibers derived from bacterial cellulose have been explored for high-performance supercapacitors, demonstrating enhanced energy storage capacity and structural stability.^252^ In potassium-ion batteries, modified PDA derivatives have been used as high-performance organic anodes, improving charge storage and cycling stability.^253^ Furthermore, PDA-coated vanadium disulfide has been explored for “fast-charging” lithium-ion batteries, offering high specific capacities and extended cycle life.^254^

Chen *et al.*^255^ demonstrated that PDA coatings on graphite felt electrodes improved electrochemical performance in vanadium redox flow batteries by enhancing surface wettability and catalytic activity.

#### 2.2.3 Bioremediation

PDA has been widely used in wastewater treatment due to its ability to modify membrane surfaces and enhance pollutant removal efficiency. An *et al.*^256^ reported the development of PDA-coated poly (vinylidene fluoride) (PVDF) membranes, which demonstrated improved anti-protein adhesion and antibacterial properties, making them highly effective in ultrafiltration systems for wastewater purification. Similarly, Silva *et al.*^257^ explored the modification of photocatalytic membranes with PDA, enhancing their ability to degrade oily contaminants in water treatment applications.

One key application of PDA in bioremediation is the removal of heavy metals from contaminated water sources. Zhou *et al.*^36^ reviewed recent advancements in PDA-based magnetic composites for pollutant removal, emphasizing their efficiency in adsorbing heavy metal ions. Additionally, Siciliano *et al.*^258^ used PDA-coated superparamagnetic iron oxide nanoparticles (SPIONs) to selectively remove copper(ii) ions (Cu^2+^) from aqueous solutions, demonstrating high binding affinity and magnetic separation capabilities.

Sun *et al.*^259^ further highlighted the use of PDA/metal–organic framework composites for rapid and selective heavy-metal removal, particularly targeting lead(ii) (Pb^2^) and mercury(ii) (Hg^2^) ions, offering a sustainable, regenerable approach to water purification.

PDA-based materials have also been investigated for their ability to degrade organic pollutants, including dyes, pesticides, and pharmaceuticals.^260^ Zeng *et al.*^261^ reported that PDA-coated nickel–cobalt alloy nanotubes (Ni_*x*_Co_100−*x*_) exhibited high efficiency in photocatalytic degradation of organic pollutants, providing a cost-effective and sustainable solution for wastewater treatment.

Additionally, Li *et al.*^262^ synthesized an iron-based PDA-poly (ionic liquid) composite as a heterogeneous Fenton catalyst, demonstrating excellent degradation performance for organic contaminants. This work underscores PDA's potential for catalytic bioremediation, particularly in advanced oxidation processes.

Beyond its chemical functionalities, PDA has been utilized in microbial bioremediation systems. Buscemi *et al.*^263^ developed a PDA-based biohybrid photoanode for interfacing bacteria with electrodes, significantly enhancing extracellular electron transfer for environmental applications such as hydrogen production and pollutant degradation.

Moreover, Flemma *et al.*^37^ explored using PDA-coated microalgae for the bioremediation of polychlorinated biphenyls (PCBs) in marine environments, demonstrating increased microbial resistance under harsh conditions and improved pollutant remediation efficiency.

## 3 Tannins: nature's tanning agents

**Fig. 2:**
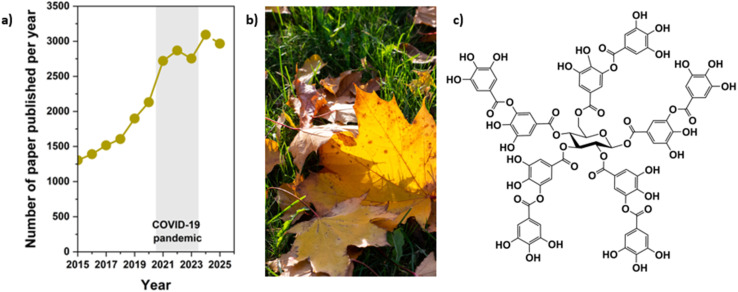
(a) Graph illustrating the total number of scientific papers published on tannins, their structures, biodegradation and their use from 2015 to 2025. The dot for the year 2025 represents papers published until December (source: *Web of Science*, 2025-12-31). (b) Image showing plant-derived biomass rich in tannins. (c) Representative chemical structure of a hydrolysable tannin.

Tannins are a diverse class of polyphenolic compounds found in plants. They are known for binding proteins and other macromolecules.^38,39^ They are essential in plant defense mechanisms, human nutrition, and industrial applications. Based on their structural composition, tannins are classified into two primary groups: hydrolysable and condensed. This review provides a detailed overview of tannin structures, their classification, and chemical properties (Fig. 2).

The upward trend in publications from 2015 to 2025 reflects a growing interest in tannins, driven by their relevance to structural characterization, biodegradation, and emerging applications in sustainable materials. Their natural origin is illustrated by the leaf biomass image, while the hydrolysable tannin structure highlights the poly-galloylated glucose core responsible for their strong reactivity, antioxidant activity, and metal-binding behaviour. When compared with polydopamine (PDA), tannins show a similarly steady rise in research output, although PDA exhibits a sharper increase due to its controlled synthetic formation and widespread use as a universal coating material. Overall, tannins provide a diverse, bio-derived alternative, whereas PDA offers a more reproducible, bioinspired platform.

### 3.1 Classification of tannins

Tannins can be generally divided into two main groups based on their structural characteristics.

#### 3.1.1 Hydrolysable tannins

Hydrolysable tannins (HTs) are esters of gallic acid (3,4,5-trihydroxybenzoic) or ellagic acid (2,3,7,8-tetrahydroxy[1]benzopyrano[5,4,3-cde][1]benzopyran-5,10-dione) with a glucose or polyol core.^40^ Acids or enzymes can efficiently hydrolyze them into simpler phenolic acids and sugars.^41^ The two major subtypes are:

(i) Gallotannins consist of multiple gallic acid units linked to central sugar, commonly glucose. They exhibit potent antioxidant and metal-chelating properties.^264^

(ii) Ellagitannins represent the largest known group of tannins. They are commonly found across various plant families and have been identified in over 500 natural compounds. These HTs contain ellagic acid units derived from oxidative coupling of gallic acid.^265^

#### 3.1.2 Condensed tannins (proanthocyanidins)

Condensed tannins (CTs) are oligomeric or polymeric flavonoids composed of catechin and epicatechin units linked by carbon–carbon bonds.^41^ They are resistant to hydrolysis and are widely found in plant-based foods and forages.

(i) Procyanidins are mainly composed of catechin [(2*R*,3*S*)-2-(3,4-dihydroxyphenyl)-3,4-dihydro-2*H*-chromene-3,5,7-triol] and its *cis*-configurated isomeric epicatechin [(2*R*,3*R*)-2-(3,4-dihydroxyphenyl)-3,4-dihydro-2*H*-chromene-3,5,7-triol] monomers.

(ii) Prodelphinidins contain gallocatechin [(2*S*,3*R*)-2-(3,4,5-trihydroxyphenyl)-3,4-dihydro-2*H*-chromene-3,5,7-triol] and epigallocatechin [(2*R*,3*R*)-2-(3,4,5-trihydroxyphenyl)-3,4-dihydro-2*H*-chromene-3,5,7-triol] units.

(iii) Propelargonidins include afzelechin [(2*R*,3*S*)-2-(4-hydroxyphenyl)-(3,4-dihydro-2*H*-chromene-3,5,7-triol)] and epiafzelechin [(2*R*,3*R*)-2-(4-hydroxyphenyl)-3,4-dihydro-2*H*-chromene-3,5,7-triol] monomers.

CTs exhibit significant biological activity, including antioxidant, antimicrobial, and protein-binding properties, influencing their use in food, medicine, and environmental applications.^266,267^ Char, an important wood-based product, yield is higher in condensed tannins than hydrolysable tannins as it has a condensed structure.^268^

### 3.2 Enzymatic degradation

Enzymatic degradation of tannins, particularly through microbial and plant-derived enzymes, has emerged as an effective approach for breaking down tannins into bioactive and environmentally friendly byproducts. The primary enzymes responsible for tannin degradation include tannase (tannin-acyl hydrolase), laccase (polyphenol oxidase), and gallate decarboxylase. These enzymes facilitate the breakdown of hydrolysable and condensed tannins into simpler phenolic compounds, improving their bioavailability and reducing their environmental impact.

#### 3.2.1 Tannase

Tannase is the most extensively studied enzyme for tannin degradation. It catalyzes the hydrolysis of esters and depsides (condensed phenols) in gallotannins and ellagitannins, releasing gallic acid (3,4,5-trihydroxybenzoic acid) and glucose.^42^ This enzyme is commonly produced by filamentous fungi (molds), such as *Aspergillus niger* and other endophytes, such as the Gram-negative bacterium *Herbaspirillum camelliae*.^228^ and has been utilized in the food and pharmaceutical industries. *A. niger* degrades gallotannins effectively and produces ellagic acid in submerged and solid-state fermentation. In industrial applications, tannase is widely used in tea production to reduce bitterness, enhance antioxidant capacity, and improve flavor.^42^

#### 3.2.2 Laccase

Laccases are multi-copper oxidases that catalyze the oxidation of phenolic compounds, leading to the breakdown of tannins. This enzyme has been intensively studied for its role in tannin degradation and environmental bioremediation.

(i) *Microbial laccase*: *Acinetobacter pittii* has been identified as a bacterial isolate capable of producing extracellular laccase, leading to the biodegradation of phenolic tannins in wastewater.^43^

(ii) *Fungal laccase*: *Trichoderma aureoviride* produces laccase that facilitates tannin degradation in leather-processing wastewater, showcasing its economic, industrial relevance.^44^

#### 3.2.3 Gallate decarboxylase

This enzyme works alongside tannase to further metabolize tannins by decarboxylating gallic acid into pyrogallol (benzene-1,2,3-triol), a multi-functional, redox-active phenolic compound. *Lactiplantibacillus plantarum* (formerly *Lactobacillus plantarum*) has been identified as a key bacterial species that uses gallate decarboxylase to efficiently degrade food tannins.^45^

### 3.3 Applications of tannins

Tannins play a significant role as raw materials in sustainable green industries. They are extensively used in leather tanning, animal feed, fisheries, and beverages. Tannins act as natural antioxidants, metal chelators, and lipid peroxidation inhibitors, making them suitable for use in pharmaceuticals and nutraceuticals.^269^ Their antimicrobial, antiviral, anticarcinogenic, and anti-inflammatory properties further enhance their utility in human health and food industries.^270,271^

#### 3.3.1 Pharmaceutical and biomedical uses

Tannins display a wide spectrum of biological, pharmacological, and biotechnological activities, and recent studies highlight the importance of viewing these effects through their shared mechanistic foundations, rather than as isolated examples. Their high phenolic content, metal-chelating capacity, and antioxidant/redox activities underpin many of their biomedical roles. Condensed tannins extracted from green-mature bananas have demonstrated potential in mitigating the toxicity of glyphosate [*N*-(phosphonomethyl glycine)]^46^ and in interacting with biologically significant metal ions.

Similarly, condensed tannins derived from green- and mature-green apples have been shown to effectively regulate cholesterol levels.^47^ Condensed tannins extracted from the bark of the wampee tree (*Clausena lansium*) exhibit novel α-glucosidase and tyrosinase inhibitory activities, making them potential candidates for treating skin diseases and as anti-diabetic agents.^48^

Tannins exhibit antibacterial, antifungal, and anticancer properties, making them promising candidates for drug development. Recent studies highlight their use in cancer prevention and therapy, particularly for inhibiting cancer cell proliferation and enhancing the efficacy of chemotherapeutic agents.^49,50^

Recent advances (2024–2026) further synthesize these findings by showing that tannins, particularly hydrolysable and condensed types, exhibit multimodal therapeutic behavior including antimicrobial, anti-inflammatory, antiviral, and anticancer properties arising from their ability to modulate oxidative stress, bind biologically relevant metal ions, and disrupt microbial or cancer-associated pathways.^51^ These reviews emphasize that tannins exert convergent mechanisms across disease models, such as scavenging reactive oxygen species, inhibiting key metabolic enzymes, destabilizing pathogenic membranes, and synergizing with chemotherapeutic agents. Together, the earlier examples and recent mechanistic insights position tannins not just as isolated bioactive extracts but as a coherent class of polyphenolic modulators with broad biomedical relevance.

#### 3.3.2 Environmental bioremediation

Tannins contribute to soil health by affecting cation exchange capacity and nitrogen retention. Their repeated application in soils has been shown to reduce soil nitrogen solubility, thereby improving nutrient retention and reducing leaching in agricultural systems. Additionally, tannins are used in bioremediation processes for detoxifying industrial wastewater, including that from the tannery industry.^52,53^

Condensed tannins have emerged as effective natural coagulants for water treatment, with chemically modified tannins from cashew, *Anacardium occidentale* and *Brazilian sabia*, *Mimosa caesalpiniifolia* demonstrating high turbidity removal efficiency, meeting international potability standards at optimal concentrations.^54^ Similarly, tannins from Norway spruce, *Picea abies* have shown enhanced water clarification when modified through aminomethylation, highlighting their potential in industrial applications.^55^ Recent studies have explored tannin-based coagulants extracted from the bark of the southern blue gum, *Eucalyptus globulus*, emphasizing sustainable production *via* microwave-assisted extraction.^56^ Additionally, tannin-based coagulants from Australian black wattle trees, *Acacia* spp. exhibited high efficiency in treating slaughterhouse effluent, further showcasing the eco-friendly advantages of tannins over conventional coagulants.^57^ The chelating properties of tannins are particularly beneficial for removing heavy metals from wastewater, making them a promising solution for environmental remediation.^58^

#### 3.3.3 Food and beverage industry

Tannins are used in food preservation due to their antioxidant and antimicrobial properties. In the wine industry, tannins significantly contribute to wine quality.^59–61^ Hydrolysable tannins, abundant in polyphenols and antioxidants, play a crucial role in enhancing wine quality.^59^ Oenological tannins help protect the color and stability of rosé wines, offering an alternative to sulfites in bio-protection strategies.^62^

Additionally, tannins are used to develop complementary foods with enhanced nutritional profiles, particularly to combat malnutrition in developing countries.^63^ Quebracho tannins (for example from red quebracho, *Schinopsis lorentzii* or white quebracho, *Aspidosperma quebracho-blanco*) and chestnut (*Castanea* spp.) tannins in cow diets were also used to increase milk protein and decrease nitrogen content in urine.^64^

#### 3.3.4 Materials science

Tannins continue to attract significant interest as sustainable, multifunctional corrosion inhibitors, owing to their strong phenolic chelating ability, redox activity, and capacity to form adherent surface films. Instead of functioning as isolated case studies, recent work reveals shared mechanistic themes across plant-derived tannins: (i) metal chelation through phenolic and galloyl groups, (ii) adsorption-based passive film formation, and (iii) synergistic interactions with metal ions or oxide surfaces. Recent studies highlight that tannic acid and mimosa tannins provide effective corrosion protection through anodizing treatments, forming stable anodic films that interact with metal surfaces to prevent degradation.^65^ Sulfonated tannins are employed to prevent scale formation in cooling water systems and boilers, while tannin-based coatings serve as primers, undercoats, and topcoats for metal surface protection.^66^ The hydroxyl groups in hydrolysable tannins act as oxygen scavengers, forming a protective adsorption layer on metal surfaces and enhancing corrosion inhibition efficiency.^67^ Additionally, tannin extracts from the mangrove, *Avicennia marina* and the Assyrian plum, *Cordia myxa* have shown high inhibition efficiency in carbon steel alloys, presenting a sustainable alternative to synthetic inhibitors.^66^

These trends are strongly reinforced by recent 2025 studies, which provide mechanistic and performance-level validation. For example, zinc-tannate pigments synthesized from *Tara* pods were shown to outperform conventional anticorrosive pigments due to their coordinated metal–polyphenol complexes and robust barrier-forming capability.^68^ Similarly, mimosa tannin-loaded ZnO smart inhibitor carriers enhanced inhibition performance in epoxy coatings by releasing tannins in response to corrosive conditions.^69^ Tannin@biochar composites used in zinc-rich coatings improved long-term anticorrosion by combining conductive pathways with tannin-mediated chemical inhibition, and tannic-acid/benzoxazine ZnO nanocomposites provided durable anticorrosion through synergistic adsorption and nanoparticle dispersion effects.^70^ Together, these findings demonstrate that tannins consistently function through coordinated adsorption, metal-complexation, barrier enhancement, and controlled inhibitor release across different material systems.

Beyond corrosion protection, tannins maintain an important role in bio-based leather tanning, offering environmentally compatible alternatives to chromium-based processes. Traditional examples such as the use of *Trema orientale* bark tannins to enhance collagen cross-linking and mechanical strength,^71^ tannins from *Caesalpinia sappan* with high polyphenolic content^72^ and tannins obtained from coffee pulp supporting sustainable tanning practices reflect a broader, consistent mechanism of collagen stabilization *via* multiple hydrogen-bonding and phenolic cross-linking interactions.^73^

## 4 Lignins: nature's wood glue

**Fig. 3:**
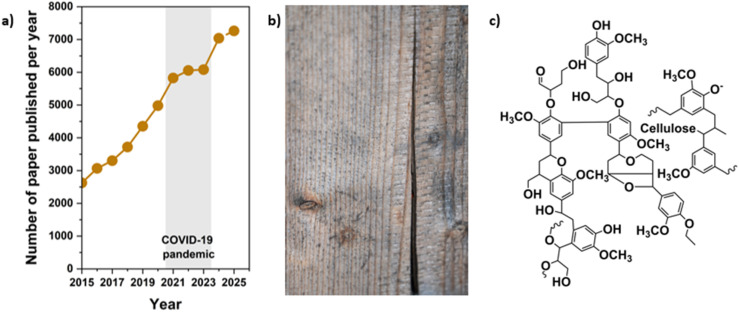
(a) Graph illustrating the total number of scientific papers published on lignins, their structures, biodegradation, and their use from 2015 to 2025. The dot for the year 2025 represents papers published until December (source: *Web of Science*, 2025-12-31) (b) Image showing lignocellulosic wood biomass that serves as a major natural source of lignin. (c) Representative chemical structure highlighting the complex, heterogeneous architecture of lignin linked to cellulose.

The publication trend from 2015 to 2025 shows a strong and continuous rise in lignin-related research, reflecting the growing interest in understanding its structural complexity, biodegradation behavior, and potential for conversion into high-value materials. The image of wood biomass highlights lignin's origin as a major component of lignocellulosic tissues, while the representative structural motif illustrates its heterogeneous, crosslinked aromatic network that is tightly associated with cellulose. When compared with polydopamine and tannins, lignin exhibits the highest publication volume, indicating broader engagement from both fundamental and applied research communities. Although PDA and tannins share polyphenolic features, lignin's abundance, structural diversity, and relevance to biomass valorization have driven a more rapid expansion in research output. Together, these trends underscore lignin's central role in sustainable materials development and its importance compared with other polyphenolic systems (Fig. 3).

Lignins are structurally complex, most abundant non-carbohydrate heterogeneous biopolymers integral to plant cell walls, contributing to mechanical strength, rigidity, hydrophobicity, and defense against microbial degradation.^74,75^ It constitutes approximately 20–30% of lignocellulosic biomass,^76,77^ and is the most abundant renewable source of aromatic compounds.^78^ A comprehensive understanding of lignin's molecular architecture, enzymatic degradation pathways, and diverse applications is essential for optimizing biorefinery processes and promoting sustainable material innovations.^79,272^ Lignin is primarily composed of three monolignols, namely paracoumaryl alcohol (4-[(1*E*)-3-hydroxyprop-1-en-1-yl]phenol), coniferyl alcohol (4-[(1*E*)-3-hydroxyprop-1-en-1-yl]-2-methoxyphenol), and sinapyl alcohol (4-[(1*E*)-3-hydroxyprop-1-en-1-yl]-2,6-dimethoxyphenol). These monolignols polymerize through radical coupling, forming complex linkages such as β–O–4′, β–5′, β–β′, and 5–5′ bonds.^273,274^ The variability in these linkages contributes to lignin's recalcitrance, making its depolymerization a significant challenge in bioconversion processes.^79^ Additionally, lignins are covalently bound to carbohydrates, forming lignin–carbohydrate complexes (LCC). Due to the plant cell wall's intricate and rigid structure, various extraction techniques have been developed to isolate lignin from diverse natural sources, including softwood, hardwood, and herbaceous biomass.^275^ Based on the isolation method, lignins can be categorized into different types, such as kraft lignin, organosolv lignin, alkali lignin, and lignosulfonate.^276^ Recent studies have utilized advanced techniques like nuclear magnetic resonance (NMR) spectroscopy, mass spectrometry, and computational modeling to elucidate lignins' structures and reactivity in biorefinery processes. These approaches have provided insights into the chemical transformations occurring during lignin extraction and processing.^79^

### 4.1 Enzymatic degradation

Fungal and bacterial oxidative enzymes, including laccases, lignin peroxidases, manganese peroxidases, and versatile peroxidases, predominantly facilitate lignin degradation. These enzymes initiate the oxidative cleavage of phenolic and non-phenolic lignin subunits, depolymerizing smaller, value-added compounds such as aromatics and organic acid.^272^

#### 4.1.1 Laccases

Laccase are multi-copper oxidases that catalyze one-electron oxidation of lignin, forming phenoxy radicals that facilitate depolymerization.^80–82^ Chen *et al.*^83^ analyzed the roles of the multi-copper oxidase-like proteins SKU5 (skewed 5) and SKS1 (SKU5-similar 1) in root cell wall formation, demonstrating their involvement in apoplastic redox reactions and modulation of reactive oxygen species. Wu *et al.*^84^ conducted a comparative genomic analysis of fungal laccases and multicopper oxidases, highlighting their diversity and potential for lignin degradation. Ahmed *et al.*^85^ reported the formation of phenoxy-type radicals from phenol adsorption on copper/iron oxides, providing mechanistic insights into radical-mediated oxidation pathways relevant to lignin degradation. Tang *et al.*^86^ introduced a manganese-nucleotide laccase-mimicking nanozyme with enhanced catalytic activity and stability, demonstrating efficient lignin model compound degradation. Dong *et al.*^87^ reviewed laccase-mediated pollutant degradation, emphasizing its role as a versatile biocatalyst in lignin oxidation pathways. Yang *et al.*^88^ presented a co-immobilized laccase-mediator system in poly(vinyl alcohol) hydrogels, enhancing laccase stability and efficiency in dye and lignin model compound degradation. These recent studies advance the understanding of multi-copper oxidases and their mechanisms in lignin oxidation, highlighting innovations in enzyme engineering and catalytic applications.

#### 4.1.2 Lignin peroxidases (LiPs)

Heme-containing peroxidases capable of oxidizing non-phenolic lignin units with high redox potential.^89,90,272^ Amara *et al.*^91^ characterized two recombinant heme-containing peroxidases, TvDyP1 and TvVP2, from turkey tail mushrooms, *Trametes versicolor*, highlighting their stability under various pH conditions and their ability to oxidize complex substrates, including lignin. Qin *et al.*^92^ explored the oxidation of non-phenolic lignin model compounds by manganese peroxidases from *Irpex lacteus* fungi, providing insights into carboxylate and radical-mediated oxidation mechanisms. Aboelnga^93^ investigated the compound formation mechanism in cytochrome c (Cyt c) peroxidase, revealing quantum chemical insights into the peroxidase catalytic cycle.

#### 4.1.3 Manganese peroxidases (MnPs)

Enzymes that generate manganese(iii) ions (Mn^3+^) to oxidize lignin phenols, enhancing delignification.^94^ Recent research highlights various enzymes, particularly manganese peroxidases (MnPs), that generate Mn^3+^ ions to oxidize lignin phenols, enhancing delignification through oxidative cleavage and depolymerization. Ley *et al.*^95^ characterized marine bacterial consortia capable of degrading lignin through the action of manganese peroxidase and other ligninolytic enzymes, showcasing the formation of low-molecular-weight lignin derivatives *via* manganese(iii)-mediated oxidation.

Wang *et al.*^96^ demonstrated the application of thermostable recombinant laccase from the hairy turkey tail, *Trametes hirsuta* in lignin degradation, emphasizing the role of Mn^3+^ ions as diffusible mediators in phenol oxidation and lignin breakdown. Jacqueline and Velvizhi^97^ reported significant delignification of fruit peels through combined physicochemical treatments, highlighting the contribution of manganese peroxidase-generated Mn^3+^ ions in breaking lignin's aromatic structures. Shi *et al.*^31^ examined the photocatalytic behavior of Mn^3+^ ions in lanthanum ferrite (LaFeO_3_) for CO_2_ reduction, providing insights into the electron transfer and oxidation processes facilitated by Mn^3+^, which are analogous to those in lignin oxidation by MnPs.

This growing body of research underscores the importance of Mn^3+^ ions generated by enzymes, such as manganese peroxidases, in lignin oxidation, thereby contributing to more efficient delignification processes.

#### 4.1.4 Versatile peroxidases (VPs)

Hybrid peroxidases combine the catalytic functions of both LiP and MnP, facilitating the oxidation of a broader range of lignin subunits.^98^ Versatile peroxidases (VPs) are a class of hybrid ligninolytic enzymes that integrate the catalytic functions of LiPs and MnPs, enabling the oxidation of a wide range of lignin subunits. Due to their multiple catalytic sites, these enzymes exhibit a unique ability to degrade phenolic and non-phenolic lignin structures.

Barber-Zucker *et al.*^277^ engineered VPs using sequence-based design, resulting in enzymes with broad substrate specificity and high environmental tolerance, making them suitable for industrial lignin valorization. Kim^278^ emphasized the catalytic potential of mushroom ligninolytic enzymes, including VPs, in converting lignin to bio-based chemicals by oxidizing both aliphatic and aromatic lignin subunits. Similarly, Civzele *et al.*^98^ demonstrated the effectiveness of VPs from *Irpex lacteus* and smoky polypore, *Bjerkandera adusta* in biomass lignin pretreatment and oxidation of ABTS [2,2′-azino-bis(3-ethylbenzothiazoline-6-sulfonic acid)]. Expanding on these findings, Granja-Travez *et al.*^279^ highlighted the diverse oxidation mechanisms of VPs through functional genomic analysis of bacterial lignin degraders, underscoring their catalytic potential beyond fungal systems. Additionally, Chen *et al.*^280^ reported that natural syringyl mediators enhance VP activity, accelerating β–O–4′ bond cleavage and the oxidation of the carbon atoms in α-position during lignin degradation. Kumar *et al.*^281^ reviewed the dual oxidation pathways of VPs from white rot fungi, which combine LiP- and MnP-like activities, enabling efficient textile dye degradation. These enzymes work synergistically to cleave the robust lignin structure, making it more accessible for microbial metabolism.

Microorganisms also play a crucial role in lignin biodegradation by producing extracellular ligninolytic enzymes:

• White-rot fungi, such as *Phanerochaete chrysosporium*, secrete a combination of LiP, MnP, and laccase to degrade lignin efficiently while leaving cellulose intact.^282^

• Brown-rot fungi, such as *Rhodonia placenta*, modify lignin through Fenton chemistry, generating hydroxyl radicals that disrupt lignocellulose structure, facilitating cellulose utilization.^79^

• Ligninolytic bacteria, including *Pseudomonas*, *Sphingomonas*, and *Rhodococcus* species, utilize diverse pathways, including oxygenase-mediated cleavage and aromatic ring fission, for lignin depolymerization.^283,284^

Recent studies highlight that microbial consortium exhibit enhanced lignin degradation through synergistic enzymatic activity, resulting in an improved yield of lignin-derived aromatic compounds.^96,285,286^

#### 4.1.5 Advances in enzymatic lignin depolymerization

Expanding the potential of lignin bioprocessing, research efforts are focused on optimizing enzymatic lignin degradation through various innovative strategies:

(i) *Deep eutectic solvents (DES)*: laccase-catalyzed lignin depolymerization in DES media has shown promise in overcoming lignin's recalcitrance by improving enzyme stability and substrate accessibility.^82^

(ii) *Metabolic engineering*: engineered microbial strains with enhanced ligninolytic enzyme production have been developed to improve lignin degradation efficiency, yielding high value bioproducts such as vanillin and phenolic compounds.^272^

(iii) *Alkali-tolerant enzymes*: recent discoveries of alkali-resistant ligninolytic bacteria have demonstrated improved lignin solubility and degradation efficiency in alkaline environments.^94^

(iv) *Synergistic enzyme systems*: the combination of peroxidases and laccases has been found to enhance lignin degradation rates, suggesting that enzyme cocktails may be key to industrial lignin valorization.^98^

### 4.2 Applications of lignins

#### 4.2.1 Material science and polymer applications

Lignin has been widely explored as a renewable precursor for the development of lignin-based polymers, adhesives, and composites, offering a sustainable alternative to fossil-derived materials.^75^

Lignin is gaining interest in additive manufacturing, particularly in 3D printing applications, where it can serve as a bio-based feedstock for fabricating complex structures.^99^ Lignin-based materials have been shown to meet essential printing requirements, including optimal printability, mechanical strength, biodegradability, biocompatibility, tissue biomimicry, and non-cytotoxicity, making them highly suitable for advanced applications such as hydrogel bio-inks in 3D bioprinting.^100^ micro-extrusion, and laser-assisted bioprinting techniques for effective tissue engineering in regenerative medicine.

Moreover, lignin-derived hydrogels have been explored for their stimuli-responsive properties, making them suitable for specialized applications in coatings and adhesives.^101^ This involves the fabrication of photonic lignin, which exhibits tunable structural color and stimuli-responsiveness. This material has been designed for wearable optical devices, innovative packaging, and advanced cosmetics due to its biocompatibility and UV resistance.^102^ In coatings and adhesives, lignin-functionalized epoxy composite coatings demonstrate UV resistance, anti-aging properties, and corrosion resistance, making them promise for metal protection and harsh environments.^103^ Lignin-based microcapsules have been developed for stimuli-triggered release applications, with studies showing that their stability can be controlled by pH and ionic strength changes.^104^

Additionally, lignin-powered bioelastomers have emerged as photothermal materials capable of converting light energy into heat, making them suitable for applications in soft robotics, smart textiles, and photothermal antibacterial treatments.^105^

#### 4.2.2 Energy storage and conversion

Lignin's high carbon content makes it an excellent precursor for the production of sustainable electrode materials. Lignin-derived porous carbons have been developed for supercapacitors, sodium-ion batteries, and fuel cells, demonstrating enhanced energy storage efficiency.^106^ Research has also focused on lignin-derived electrode materials for supercapacitors, which provide high surface areas and tunable porosity, essential for advanced energy applications.^107^ Reenu *et al.*^108^ provided a comprehensive review of advancements in electrode materials, emphasizing the role of lignin-derived carbons in enhancing supercapacitor energy storage performance.

Li *et al.*^109^ introduced a Fenton reaction-based activation method for lignin-derived porous carbons, resulting in an exceptional specific surface area and high cycling stability in supercapacitors. Zhang *et al.*^110^ further highlighted the potential of lignin as a sustainable precursor for high-performance porous carbon electrodes suitable for both supercapacitors and sodium-ion batteries.

In the bioenergy sector, lignin has been extensively investigated as a potential feedstock for biofuels and biochemicals. The “lignin-first” biorefinery approach has emerged as a promising strategy for lignin utilization, emphasizing its valorization alongside carbohydrate processing. This method enhances the selective production of high-value aromatic monomers, which serve as essential precursors for biofuels and chemicals.^75^ Unlike conventional approaches, which often lead to significant lignin degradation and underutilization, the “lignin-first” strategy focuses on preserving lignin's structural integrity while maximizing conversion efficiency.^107^ Lignin valorization efforts focus on producing high-value chemicals such as phenols, aromatics, and bio-based resins. Catalytic and enzymatic depolymerization strategies are being refined to improve lignin conversion efficiency into useful fine-chemical intermediates.^111^

The catalytic depolymerization of lignin into valuable chemicals, including aromatic hydrocarbons and bio-based fuels, has been a major research focus.^81^ Wang *et al.*^96^ (2024) demonstrated the efficacy of nano-architectured membranes with tailored ion transport channels, achieving high-efficiency osmotic energy harvesting. Fang *et al.*^112^ also explored the potential of ultrathin covalent organic framework membranes for harvesting osmotic energy from organic solutions, achieving remarkable power output from salinity gradients.

These studies collectively highlight the dual potential of lignin-derived materials to advance energy storage and renewable energy harvesting technologies.

#### 4.2.3 Environmental applications

Lignin has exceptional potential for the development of functional hydrogels.^113^ Recent research has shown that lignin-based hydrogels exhibit high strength, anti-freezing properties, and rapid polymerization, making them highly versatile for a range of industrial and environmental applications.^114^

Another use of lignin-based hydrogels is their ability to effectively remove copper from water and soil through adsorption and passivation, offering an eco-friendly approach to soil remediation.^115^ Moreover, lignocellulosic hydrogel adsorbents exhibit enhanced heavy-metal binding capacity due to the structural support provided by lignin, making them superior to traditional adsorbents.^116,117^ In addition to its renewable and biodegradable nature, lignin has intrinsic metal-ion chelation properties, making it particularly suitable for environmental pollution control applications.^118^

Using lignin-based materials as carbon dioxide adsorbents was demonstrated, contributing to carbon capture technologies,^119^ and reducing greenhouse gas emissions.^120^ Lignin-derived bio-adsorbents are being extensively studied for wastewater treatment.

Furthermore, lignin has shown potential in the food packaging industry, and it can be used to improve the mechanical and UV-blocking properties of packaging films.^121^ Lignin-based nanocomposites have been explored for antimicrobial coating applications, offering new solutions for food preservation.^122,123^

Lignin can be integrated either by blending free lignin with the desired polymer or by utilizing lignin nanoparticles. Chromophores, such as carbonyl and conjugated phenol groups, within the lignin structure allow this polymer to absorb light in the UV range (200–400 nm). This property offers an additional advantage: lignin fillers in food packaging help protect food from UV radiation.^110,124^

#### 4.2.4 Agriculture applications

Lignin has been utilized in various agricultural applications, including fertilizer, pesticide, and plant growth regulator.^125,126^ Abbas *et al.*^127^ demonstrated that lignin-based pesticides can significantly reduce soil leaching, soil pollution, and groundwater contamination. Additionally, lignin exhibits slow-release properties, is naturally abundant, and has eco-friendly chemical characteristics, making it a suitable candidate for pesticide applications.^128^

Yu *et al.*^129^ reported that lignin-based nano/microcapsules offer targeted, pH-responsive pesticide delivery with enhanced adhesion and reduced environmental toxicity. Wang *et al.*^96^ highlighted the value of lignin-based intelligent release systems, which improve pesticide efficacy while lowering environmental impact. Furthermore, Zheng *et al.*^130^ noted that lignin-enhanced formulations promote plant growth and remove persistent organic pollutants such as polycyclic aromatic hydrocarbons (PAHs) and dichlorodiphenyldichloroethylene (DDE) from contaminated soils.

Gigli *et al.*^131^ discussed the role of lignin nanoparticles in controlled pesticide release, enhancing plant uptake efficiency while minimizing environmental losses. Liu *et al.*^286^ found that lignin-based coatings can improve the slow-release performance of fertilizers, suggesting a similar advantage for pesticide delivery.

These findings underscore the significant potential of lignin-based materials in sustainable agriculture, offering enhanced pesticide performance and a reduced ecological impact.

#### 4.2.5 Biomedical applications

Across biomedical formats (films, fibers, foams, and hydrogels), lignin's dense phenolic architecture enables multimodal interactions, including hydrogen bonding, π–π stacking, and metal-ion coordination/reduction, so it can both reinforce polymer networks and modulate the biological microenvironment. At the matrix level, these interactions increase crosslink density and cohesion and can create adhesive, injectable, or conductive networks when combined with complementary polymers; at the biological level, lignin's antioxidant and antimicrobial activities mitigate excessive ROS and infection two universal barriers to tissue regeneration. These properties are widely documented in recent 2025 syntheses of lignin-based hydrogels and biomaterials and in materials chemistry reviews describing how controlled (re)polymerization/functionalization of technical lignins improves performance in advanced composites.^132–134^

The versatile biological properties of lignin have enabled its integration with organic and inorganic polymers to develop lignin-based composites for bone engineering applications. For instance, Luzi *et al.*^135^ explored the effects of PLA and lignin/tin oxide films on human bone regeneration. Their study demonstrated that human bone mesenchymal stem cells (hbMSCs) and adipose stem cells successfully adhered to the film, with vinculin focal adhesion spots observed on all PLA films, indicating strong hbMSC interaction with the matrices.

Abudula *et al.*^136^ recently crosslinked lignin with gelatin to create antibacterial, antioxidant, and injectable cryo-gels that support bone cell differentiation. Their findings revealed that gelatin containing 0.2% lignin exhibited a compression modulus and compression stress that were 1.8 and 7 times higher, respectively, than those of pure gelatin gels. Cell experiments further confirmed that the lignin-based cryo-gels had low activation effects on mouse bone marrow-derived dendritic cells and displayed excellent biocompatibility. Recent 2025 overviews of tissue-engineering scaffolds emphasize that incorporating antioxidant, antibacterial, and sustainable biopolymers, particularly lignin, offers a powerful strategy for stabilizing the redox and inflammatory milieu while simultaneously reinforcing the structural matrix. Across current reviews of functional and 3D-printed bone scaffolds, lignin is consistently highlighted as a multifunctional phase capable of modulating oxidative stress, tempering inflammation, and synergizing with osteoconductive ceramic components. Accordingly, a key design rule emerging from these analyses is to integrate lignin as either a dispersed filler or a network co-former so that mechanical integrity is coupled with ROS and inflammation control, ultimately supporting osteogenesis and angiogenesis within polymer–ceramic composite scaffolds.^137–139^

Amini *et al.*^140^ used polycaprolactone and lignin as feedstocks to fabricate an electrospun fiber conduit for sciatic nerve regeneration in rats. The *vivo* study revealed that rats implanted with a 15% lignin-containing scaffold showed no significant differences compared with those receiving autografts in nerve function, conduction velocity, and expression of cell differentiation markers. These findings support the conclusions of Saudi *et al.*^141^, further highlighting the promising potential of lignin-based composites for neural tissue engineering.

Films and hydrogels are the most widely used technologies for developing lignin-based wound-healing composites for medical applications. For instance, Pérez-Rafael *et al.*^142^ employed a hydrogel containing lignin-capped silver nanoparticles, which exhibited potent antioxidant properties and strong antibacterial activity against *Staphylococcus aureus* and *Pseudomonas aeruginosa* when applied to chronic wound surfaces.

Alqahtani *et al.*^143^ demonstrated that lignin nanoparticles loaded with curcumin [(1*E*,6*E*)-1,7-bis(4-hydroxy-3-methoxyphenyl)hepta-1,6-diene-3,5-dione] possess potent antibacterial activity against Gram-positive bacteria, particularly *Staphylococcus aureus*, a common pathogen in infections. Their study found that the lignin-curcumin formulation reduced *S. aureus* by 95.4%, highlighting its potential as an effective antimicrobial treatment. Building on these scaffold-level principles, 2025 studies demonstrate how lignin operationalizes these mechanisms within advanced wound dressings. Lignin-sulfonate/PPy conductive hydrogels incorporating AgNPs use lignin as both dispersant and dopant, achieving antibacterial rates above 90% against *E. coli* and *S. aureus*, while supporting hemostasis, tissue adhesion, and when paired with mild electrical stimulation approximately 94% wound closure by day 14 with enhanced collagen deposition and re-epithelialization.^144^ Similarly, PVA-chitosan/lignin@CdZnO hydrogels leverage lignin as a bioactive network co-former and nanoparticle mediator, yielding markedly improved bactericidal and antioxidant performance and ∼91% closure within 10 days in rat models.^145^ Bacterial-cellulose/lignin/MgO nanocomposites further illustrate lignin's versatility, providing broad antimicrobial activity (*E. coli*, *S. aureus*, *C. albicans*) while maintaining the mechanical robustness and water-handling properties required for practical dressings.^146^ Conceptually, 2025 wound-care overviews converge on the need for multifunctional, stimuli-responsive hydrogels capable of simultaneously controlling infection and oxidative stress while sustaining a pro-regenerative microenvironment, precisely the design space in which lignin's phenolic chemistry, redox activity, and capacity to reduce or stabilize metal nanoparticles *in situ* confer unique translational value.^147–149^

## 5 Melanins – nature's pigment of darkness

Over time, melanin has come to represent a diverse group of bio-pigments in most organisms. It is now broadly defined as a heterogeneous polymer, a group of complexes, recalcitrant, and pervasive polycyclic bio-polymeric skin pigments produced by specialized skin cells called *melanocytes*, primarily formed through the oxidative polymerization of intermediate phenols, quinones, and indole compounds.^150^

**Fig. 4:**
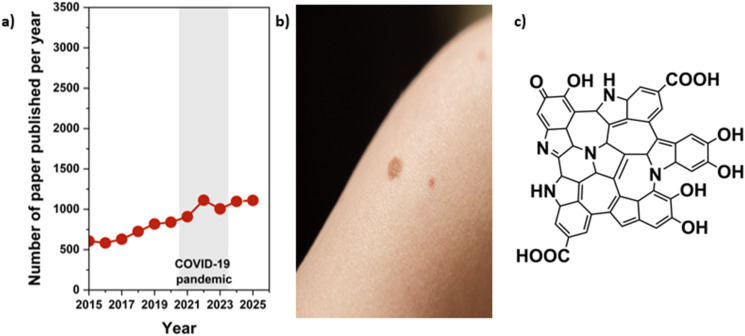
(a) Graph illustrating the total number of scientific papers published on melanins, their structures, biodegradation, and their use from 2015 to 2025. The dot for the year 2025 represents papers published until December (source: *Web of Science*, 2025-12-31). (b) Image showing melanin-containing biological tissue. (c) Representative chemical structure of melanin illustrating its indole-based, heterocyclic polymeric framework. (Structure of melanin comes from: https://doi.org/10.5935/0103-5053.20140277).

As indicated in Fig. 4, the publication trend from 2015 to 2025 shows a gradual but steady increase in melanin-related research, with annual outputs rising from fewer than 600 papers in 2015 to around 1100 papers by 2025. This growth reflects expanding interest in melanin's photoprotective behaviour, redox properties, and potential use in functional materials and biomedical applications. The image of melanin-containing biological tissue highlights its natural occurrence in organisms, while the representative chemical structure illustrates the characteristic indole-based, heterocyclic network that gives melanin its unique optical absorption, radical-scavenging capacity, and structural resilience. Compared with polydopamine, tannins, and lignin, melanin exhibits a noticeably lower publication volume, suggesting that it remains a more specialized research area. PDA exhibits the fastest growth due to its widespread use as a synthetic, mussel-inspired coating; tannins show strong interest driven by their sustainability and plant-derived polyphenolic diversity; and lignin leads in overall publications owing to its abundance and central role in biomass valorisation. Together, these trends position melanin as a promising but comparatively underexplored polyphenolic system within the broader landscape of bio-based and bioinspired materials.

Recent studies have expanded our understanding of melanin's biochemical synthesis and evolutionary significance. The biosynthetic pathway of melanin involves enzymatic oxidation, where l-tyrosine [2-amino-3-(4-hydroxyphenyl)propanoic acid] is converted into l-dopaquinone (3,4-dioxo-3,4-dihydro-l-phenylalanine). This is followed by a series of complex polymerization reactions leading to eumelanin, pheomelanin, or neuromelanin.^151^

### 5.1 Types and molecular structure of melanin

Melanin is categorized into three main types:

(i) *Eumelanin*: a black or brown pigment primarily composed of 5,6-dihydroxyindole (1*H*-indole-5,6-diol, DHI) and 5,6-dihydroxyindole-2-carboxylic acid (5,6-dihydroxy-1*H*-indole-2-carboxylic acid, DHICA), linked through oxidative polymerization.^152,153^

(ii) *Pheomelanin*: a reddish-yellow pigment that contains benzothiazine and benzothiazole derivatives, resulting from the incorporation of cysteine into dopaquinone during synthesis.

(iii) *Neuromelanin*: found in the brain; this melanin type has a unique structure incorporating catecholamines, playing a role in neuroprotection.

Melanin is synthesized through the oxidation of l-tyrosine, catalyzed by the enzyme tyrosinase, followed by polymerization into high-molecular-weight structures.^154^ Melanin's chemical composition shows mostly characteristic structural diversity.

### 5.2 Enzymatic degradation

#### 5.2.1 Peroxidase-mediated melanin degradation

Recently, reports indicated that the ligninolytic enzyme (LE) group can degrade melanin. White rot fungi have been investigated as potential candidates for natural whitening due to their ability to produce melanin-degrading LEs, which include lignin peroxidase, manganese peroxidase, and laccase. These enzymes facilitate the oxidative degradation of melanin, leading to its decolorization and depolymerization.^155^

LiP from *Phanerochaete chrysosporium* has been extensively studied for its ability to catalyze the oxidative degradation of melanin. This enzyme facilitates the breakdown of melanin polymers, leading to decolorization and solubilization.^156^

A comparative analysis of fungal and bacterial peroxidases showed that LiP from *P. chrysosporium* has the highest catalytic efficiency for non-phenolic aromatic compounds, including melanin, due to its ability to facilitate long-range electron transfer.^157^ Another study confirmed that LiP has a redox potential of *ca.* 1.33 V, significantly higher than those of bacterial dye-decolorizing peroxidases (DyPs) and fungal MnP, making it more efficient at oxidizing and degrading melanin.^158^

#### 5.2.2 Laccase-driven melanin biodegradation

A synergistic laccase-peroxidase enzyme complex enhances melanin degradation, making it highly effective for skin whitening and environmental applications.^159^ Among various laccase-producing fungal strains, white rot fungi of the *Trametes* genus have been extensively studied for their melanin degradation potential. Notably, *Trametes pubescens* JS18 exhibited 81% melanin degradation within 24 hours when supplemented with hydroxybenzotriazole (1*H*-1,2,3-benzotriazol-1-ol) as a mediator.^160^ Moreover, laccase derived from two strains of *T. pubescens* have been studied for their ability to degrade melanin. Gigli *et al.*^131^ (2022) showed that laccase from *T. pubescens* can effectively degrade eumelanin when paired with a cocktail of natural phenol redox mediators, including vanillin (4-hydroxy-3-methoxybenzaldehyde), syringaldehyde (4-hydroxy-3,5-dimethoxybenzaldehyde), acetosyringone [1-(4-hydroxy-3,5-dimethoxyphenyl)ethan-1-one], acetovanillone [1-(4-hydroxy-3-methoxyphenyl)ethan-1-one], and vanillyl alcohol [4-(hydroxymethyl)-2-methoxyphenol]. Park *et al.*^161^ reported that laccase from *T. pubescens* could break down both eumelanin analogs and natural melanin synthesized by melanoma cells. More recently, Nadhilah *et al.*^162^ highlighted that a combination of laccase and MnP produced by *T. hirsuta* effectively degrades melanin in the presence of suitable mediators, namely phenolic compounds such as gallic acid, vanillic acid (4-hydroxy-3-methoxybenzoic acid), and syringic acid, demonstrating the synergistic role of these enzymes in melanin biodegradation.

Laccase-mediated melanin decolorization has emerged as a viable alternative to peroxidases because laccase operates without hydrogen peroxide and can use natural mediators, making it an eco-friendlier solution.^163^

#### 5.2.3 Tyrosinase and its role in melanin breakdown

Tyrosinase, a key enzyme in melanin biosynthesis, is also essential in the degradation of melanin. This enzyme catalyzes the oxidation of melanin precursors, generating reactive intermediates that facilitate melanin breakdown.^164^

Recent studies have shown that tyrosinase-mediated oxidation produces quinone-based intermediates, which undergo further degradation through radical-driven reactions, resulting in the structural fragmentation of melanin polymers.^165^ Additionally, tyrosinase activity can be modulated through external factors, including flavonoid compounds, which can either enhance or inhibit its oxidative action on melanin.^166^ The presence of tyrosinase inhibitors, such as the marine-derived compound gagunin D [((1*R*,2*S*,3a*R*,4*S*,5 a*R*,6*S*,7*S*,8*S*,10 a*R*,10b*S*)-7-acetyloxy-4,6-di(butanoyloxy)-8-hydroxy-3a,5a,9-trimethyl-1-propan-2-yl-1,2,3,4,5,6,7,8,10a,10b-decahydrocyclohepta(e)inden-2-yl)butanoate], has modulated tyrosinase activity and accelerated melanin degradation. Derivatives of kojic acid [5-hydroxy-2-(hydroxymethyl)-4*H*-pyran-4-one], along with other bioactive tyrosinase inhibitors, effectively reduce melanin synthesis and promote its breakdown by targeting the active site of tyrosinase and disrupting its catalytic function.^167,168^

Natural plant-derived inhibitors such as arbutin, vanillin, and flavonoid-based compounds have been extensively studied for their anti-melanogenic properties. These compounds serve as competitive inhibitors by binding to the copper-active site of tyrosinase, preventing melanin biosynthesis and enhancing degradation pathways.^169,170^ Additionally, synthetic modifications of natural inhibitors have led to the development of more potent and stable tyrosinase-blocking agents, thereby broadening their applications in the cosmetic, pharmaceutical, and medical fields.^171^

### 5.3 Application of melanins

#### 5.3.1 Bioremediation

Melanin exhibits a high binding capacity and strong affinity for various heavy metals, including copper(ii), zinc(ii), magnesium(ii), cadmium(ii), and manganese(ii) ions making it highly effective in heavy metal sequestration and environmental remediation.^287^ The metal-binding property results from the interaction of metal ions with functional groups found in melanin, including carboxyl, hydroxyl, and amino groups, allowing it to act as a natural chelating agent in polluted environments.^150,288^ The ability of melanin to adsorb and interact with heavy metals has led to its application in bioremediation strategies, where it is employed to remove toxic metals from wastewater and recover valuable metal ions from industrial effluents.^289^ Additionally, heavy metals adsorbed by melanin can be effectively released by dissolving melanin in an alkaline environment, thus ensuring metal recovery without generating secondary pollutants.^152^ Furthermore, nanomaterials modified with melanin have enhanced their ability to scavenge metals, facilitating real-time detection and removal of heavy metals.^287^ In a separate application, nanofiber membranes infused with fungal melanin from *Armillaria cepistipes* have proven highly effective at adsorbing heavy metals from water.^290^ These membranes exhibited a greater affinity for hazardous heavy metals than for essential trace metals, making them especially useful for selective metal removal. Similarly, fungal melanin extracted from *Amorphotheca resinae* has been used for heavy-metal adsorption and has been found to be reusable, highlighting its cost-effectiveness and sustainability in environmental remediation.^291^ When metal ions were adsorbed at an optimal pH of 5, subsequent exposure to acidic conditions facilitated their desorption, enabling the regeneration and reuse of melanin multiple times. This adsorption–desorption process was repeated effectively for up to 5 cycles without significant loss in binding efficiency.

Biosynthesized melanin nanoparticles exhibit high adsorption capacities, making them suitable for industrial-scale water purification.^292^ Additionally, melanin-coated membranes have been developed for continuous filtration processes, thereby enhancing heavy metal removal efficiency in batch and continuous-flow systems.^293^

Hybrid materials combining melanin with titanium dioxide have been explored for their dual-action capability to remove both heavy metals and organic dyes from wastewater under visible light.^294^ These materials leverage melanin's adsorption properties alongside titanium dioxide's photocatalytic activity, providing a sustainable approach to degrading organic contaminants.

The ability of melanin-producing microorganisms to immobilize uranium highlights their potential role in bioremediation strategies for radioactive waste management.^295^ The use of melanin-functionalized bacterial systems for environmental detoxification and uranium recovery is gaining interest as a low-cost alternative to conventional radioactive waste treatment methods.^296^

#### 5.3.2 Biomedical field

Melanins and melanin-like polymers, combined with specific ligands, metal clusters, and oxides, are extensively utilized in biomedical applications, including imaging techniques, optoacoustic devices, and highly adhesive biomaterials.^172–174^ Rather than functioning merely as another bio-inspired coating, melanin acts as a mechanistic “hub” material whose redox activity, metal-chelating capacity, and broadband optical absorption can be modularly integrated with inorganic or organic components. Compared with other biopolymers such as chitosan, alginate, or tannin-based systems, melanin uniquely combines paramagnetism, strong NIR absorption, and intrinsic radical buffering, enabling multifunctionality that is difficult to achieve with single-function biopolymers.^175^

As drug carriers, melanin-based nanomaterials are being widely investigated for their high surface area, pH responsiveness, and ability to bind drugs and metal ions.^176–178^ Mechanistically, drug loading is governed by π–π stacking, hydrogen bonding, and metal coordination interactions that are more tunable than those in polysaccharide or protein carriers, which rely mainly on electrostatics or enzymatic degradation.^179^ Recent work using natural melanin nanoparticles for simultaneous chemotherapy and photothermal therapy demonstrates improved tumor inhibition and validates melanin's dual therapeutic role.^180^

Pharmacologically active molecules can be covalently attached to the melanin surface because of its abundance of functional groups, including 1,2-benzoquinone (cyclohexa-3,5-diene-1,2-dione), amines, catechol, and imines. Additionally, these molecules can be encapsulated within the melanin polymer matrix through non-covalent interactions, offering a versatile approach for drug delivery. It has emerged as a key biomolecule in tumor-targeted cancer therapy due to its unique biochemical properties, including a high binding affinity for certain drugs, strong optical absorption, and the ability to modulate oxidative stress. These characteristics allow for selective tumor targeting, making melanin a valuable component in precision oncology and biomaterial applications.^181^ Studies have shown that melanin-modified nanoparticles can be loaded with chemotherapeutic agents, photosensitizers, or immunomodulators, enhancing therapeutic outcomes in melanoma and other aggressive cancers.^182^ Melanin-based multifunctional nanoplatforms have also been designed for targeted drug delivery and theranostic applications.^183^ For instance, prostate-specific membrane antigen (PSMA)-targeted melanin-like nanoparticles have been developed for imaging and therapy of prostate cancer, significantly enhancing tumor localization and treatment efficiency.^184^

Melanin's strong near-infrared (NIR) optical absorption makes it an excellent candidate for photothermal therapy (PTT). When activated by NIR laser irradiation, melanin-coated nanomaterials generate localized heat, selectively ablating melanin-rich tumor cells while preserving surrounding healthy tissues.^178^ Furthermore, researchers are exploring melanin-based immunotherapies to enhance anti-tumor immune responses by leveraging melanin's antioxidant and immune-modulating properties to improve the efficacy of cancer vaccines.^185,186^ A 2025 mechanistic review of photothermal nanomaterials identifies melanin-like systems as uniquely capable of combining heat generation with redox modulation, enabling synergistic therapeutic effects.^187^ Melanin-like nanoparticles have also been engineered for photothermal-triggered drug release, enabling spatiotemporal control of therapy and demonstrating improved release kinetics under NIR irradiation.^222^ However, the balance between melanin's ROS-scavenging and immunostimulatory effects remains poorly understood and may vary with oxidation state, dose, and tumor microenvironment.

Human melanin shares structural similarities with fungal melanin, leading to the use of melanin extracted from *Cryptococcus* spp. in the development of monoclonal antibodies that specifically bind to human melanin, with potential therapeutic applications for metastatic melanoma treatment.^188^ A melanin-mediated immune mechanism against COVID-19 has been proposed, suggesting that melanin may interact with and inhibit the active site of the serine protease furin, a key enzyme required for viral entry into host cells.^189^ However, concerns have been raised about the *in silico* analysis used in this study, as polymeric eumelanin structures may not physically fit within the small furin binding site, highlighting the need for further experimental validation. In this controversial discussion, the authors also proposed broader biological activities of melanin, including antiviral, antimicrobial, anti-inflammatory, antitumor, and immunostimulatory properties.^189^

A groundbreaking study revealed that melanin precursors, including DHICA and l-3,4-dihydroxyphenylalanine (l-DA), exhibit strong interactions with the SARS-CoV-2 spike protein, indicating their potential as effective antiviral agents. These findings underscore the biological relevance of melanin derivatives in preventing viral entry and inhibiting replication.^190^

Melanin and melanin-like coatings have been utilized to promote cell attachment and proliferation, thereby facilitating the regeneration of skin, bone, and nerve tissue.^191^ For instance, melanin-integrated hydrogels and biomimetic scaffolds have been developed for wound healing applications, showing antioxidant and anti-inflammatory effects that promote faster recovery.^192^ Furthermore, melanin nanoparticles combined with mesenchymal stem cells demonstrate potential for nerve regeneration and stroke recovery by reducing oxidative stress and apoptosis.^193^

Melanin's paramagnetic properties enable its use as a contrast agent in magnetic resonance imaging (MRI) and photoacoustic imaging, thereby enhancing non-invasive diagnostics.^174^ Additionally, melanin-based biosensors have been designed to detect biomolecules, enzymes, and heavy metals, thereby expanding their application in point-of-care diagnostics.

#### 5.3.3 Food industry

Melanin is attracting increasing attention for its antimicrobial, antioxidant, and ultraviolet-resistant properties in food packaging applications. Studies have shown that melanin-based nanocomposites enhance the stability and protective function of food packaging materials, effectively blocking ultraviolet (UV) radiation and extending product shelf life.^194^ The antioxidant effect of melanin in active packaging films has been evaluated, while results showed the capacity of melanin to prevent food oxidation.^195^ A study by Bang *et al.*^196^ showed that melanin-based composite films provided strong UVB (wavelength range 280–315 nm) protection for potatoes while enhancing packaging strength, permeability, and color. These films offer an eco-friendly solution to prevent spoilage during storage and distribution. Roy *et al.*^197^ developed hardwood cellulose nanofiber-based nanocomposite films with melanin, enhancing UV shielding, mechanical strength, and antioxidant properties. The films blocked 96% of UV light.

The incorporation of melanin or melanin-like nanoparticles into various composite films has been shown to significantly enhance the mechanical strength, hydrophobicity, antioxidant activity, ultraviolet-visible light (UV-vis) barrier properties, and thermal insulation.^194,195^

Łopusiewicz *et al.*^198^ investigated a whey protein-based active packaging film incorporating melanin extracted from watermelon (*Citrullus lanatus*) seeds. Their findings revealed that adding bioactive functional materials enhanced several physical properties of the composite film. In another investigation, melanin extracted from cuttlefish (*Sepiina*) ink has been utilized to develop edible antimicrobial packaging films.^199^ A dual-mode bactericidal bilayer film composed of glucomannan [β(1→4)-d-gluco-d-mannoglycan] from konjac (*Amorphophallus konjac*), polycaprolactone, and quercetin-(3,3′,4′,5,7-pentahydroxyflavone) loaded melanin-like nanoparticles was generated to enhance food preservation and inhibit bacterial growth. This film has demonstrated strong antibacterial properties by effectively inhibiting the growth of *Escherichia coli* and *Listeria monocytogenes*.^200^

Microbial melanin production is explored as a sustainable, natural alternative to food colorants, offering non-toxic, biodegradable pigmentation.^290^ Eumelanin and allomelanin, a nitrogen-free melanin, appear black, whereas pheomelanin ranges from yellow to reddish. These melanin types' of varying quantities and relative proportions create a spectrum of colors, from black and brown to red, contributing to diverse food colorations.^201^

Melanin also exhibits excellent heat stability, ensuring its quality in food applications. The melanin extracted from *Ganoderma lucidum* demonstrated high stability within a pH range of 6.0–12.0 under natural and UV light and at temperatures between 20–80 °C.^202^ Natural melanin remained stable in the presence of typical food additives such as sugar, sodium chloride, sodium citrate, and potassium sorbate. Therefore, melanin can serve as a natural colorant in various food products, including bread and sausage, as well as popular, black-colored foods like squid (*Teuthida*) ink spaghetti and dumplings, contributing to the current trend of “black food”.^203,204^

Melanin exhibits inhibitory effects against major foodborne pathogens that cause severe human infections, suggesting its potential as a natural antimicrobial agent to enhance food storage stability. Squid ink melanin demonstrated antibacterial properties against *Escherichia coli* and *Listeria monocytogenes*, both harmful bacteria responsible for food spoilage and human diseases.^205^ Melanin extracted from date palm fruits showed antibacterial activity against Gram-positive and Gram-negative foodborne pathogens.^206^ Melanin from horsehair and watermelon seeds demonstrated significant antibacterial effects against *E. coli* and *Staphylococcus aureus*, completely inhibiting bacterial growth within four hours.^207,208^

Melanin can act as a lipid peroxidation inhibitor to enhance food storage stability, improving overall food quality. This opens the door to a new era in which melanin is utilized as a functional food ingredient.^209^

#### 5.3.4 Cosmetic industry

The cosmetic industry increasingly favors the development and use of natural active ingredients. Melanin is a nontoxic natural pigment that can be used as a cosmetic colorant. Its antioxidant and radioprotective properties make it an effective photoprotective agent, helping extend the shelf life of cosmetic products. A significant advancement in biotechnological melanin synthesis was reported by El-Zawawy *et al.*^210^ who optimized melanin production in *Streptomyces djakartensis*, highlighting its radioprotective, antioxidant, and UV-absorbing properties and suggesting substantial utility in sunscreen and anti-aging formulations. Additionally, melanin has an opsonizing effect on the skin, providing anti-aging benefits similar to those of vitamin C and E.^211^ Clinical interest in melanin's topical use was reinforced by Petrosyan and Nameq^212^ who evaluated melanin-containing formulations for the treatment of solar dermatitis. The findings indicated practical anti-inflammatory and photoprotective benefits, with minor adverse effects, further supporting melanin's integration into dermatological care.

## 6 Challenges in the utilization of PDAs, lignins, tannins, and melanins

Despite the significant potential of PDA, lignins, tannins, and melanins in various applications, several key challenges must be addressed to harness their benefits fully. These challenges span structural characterization, process optimization, scalability, enzymatic degradation control, and polymer modification.

(i) *Structural complexity and characterization*: one of the primary challenges associated with these biopolymers is their intricate and heterogeneous structures. PDA, lignins, tannins, and melanins possess highly variable compositions influenced by their source, extraction methods, and environmental conditions. This complexity makes it challenging to characterize their molecular structures and predict their physicochemical properties precisely. Advanced analytical techniques such as nuclear magnetic resonance (NMR), Fourier-transform infrared spectroscopy (FTIR), and high-performance liquid chromatography (HPLC) are used to elucidate these structures. However, developing standardized methodologies for accurate characterization remains an ongoing challenge, especially ensuring reproducibility in industrial applications.

(ii) *Process optimization for polymerization and degradation*: optimizing these biopolymers' polymerization and degradation processes is crucial for achieving desired mechanical, chemical, and functional properties. In the case of PDA, controlling oxidation-mediated self-polymerization enhances film formation and adhesion.^234^ For lignins and tannins, adjusting polymerization pathways affects their reactivity and performance in adhesives, coatings, and composite materials. Likewise, melanin biosynthesis must be optimized to attain uniform pigment properties.

(iii) *Scalability and cost-effective production*: for widespread industrial adoption, developing scalable and cost-effective production methods is essential. Although these biopolymers come from abundant natural sources such as plant extracts, fungi, and synthetic pathways, large-scale extraction and synthesis still pose economic challenges.

Large-scale PDA synthesis faces hurdles in maintaining uniform polymer properties across batches.^34^ Sustainable processing techniques that minimize waste and chemical usage are critical. For instance, lignin valorization in bio-refineries is still limited by low yield recovery and high processing costs.^121^ Biotechnological advancements, including enzyme-assisted synthesis and nanotechnology integration, are still being explored to improve cost efficiency.

(iv) *Controlling enzymatic degradation for stability*: the enzymatic degradation of these biopolymers presents both opportunities and challenges. While biodegradability is desirable for environmental applications, excessive enzymatic breakdown can compromise the stability of materials used in coatings, biomedical implants, and structural composites.^166^ Enzyme inhibitors, chemical cross-linking, and selective functionalization can help regulate enzymatic activity. For example, enzyme-resistant lignin derivatives are being explored for high-performance composite materials, while melanin-based biomaterials require stabilization techniques to prevent premature degradation. Further research into enzyme-material interactions and resistance mechanisms is necessary to improve material longevity.

(v) *Modification and functionalization for advanced applications*: expanding the functional versatility of PDA, lignins, tannins, and melanins through chemical and physical modifications unlocks new applications. Surface functionalization, grafting, and hybridization with nanomaterials can significantly enhance their properties. For example, PDA-functionalized nanoparticles improve drug delivery capabilities, tannin-based polymers are used in corrosion protection, and lignin-derived carbon materials are employed in energy storage.^174^ Advanced modification techniques, such as plasma treatment, enzymatic functionalization, and supramolecular assembly, further widen their functional potential. However, ensuring stability, biocompatibility, and environmental safety of modified polymers remains a critical consideration for commercial applications.^111^

## 7 Future perspectives: advancements in green processing technologies

Research into bioinspired polymers such as PDA, tannins, lignins, and melanins – promises a significant transformation in material science that aligns with green chemistry principles. Owing to their renewable characteristics, enzymatic degradability, and diverse structures, these polymers play a crucial role in developing sustainable alternatives to traditional petroleum-based materials. Future efforts will focus on increasing their production and tailoring their properties for specific uses in energy, healthcare, packaging, and environmental cleanup. There will also be a growing emphasis on life-cycle assessments and ecological footprint modeling to evaluate the long-term sustainability of biopolymer-based systems.^297^

A significant challenge remains the limited capacity to control molecular heterogeneity in these polymers, which impedes reproducibility, regulatory approval, and industrial standardization. Creating analytical frameworks that can decipher their hierarchical structures from monomer chemistry to supramolecular assembly will be crucial for turning laboratory breakthroughs into market-ready materials.

In materials science, these biopolymers present opportunities to engineer multifunctional composites that integrate structural strength, environmental responsiveness, and biodegradability. Material formulations derived from these polymers are increasingly being investigated for advanced functionalities such as photothermal conversion, anti-corrosion coatings, and electrically conductive composites. Incorporating these bioresources into engineered nanostructures and hierarchical assemblies may revolutionize lightweight construction materials, implantable devices, and green electronics.^298^

However, the field still lacks reliable predictive rules for adjusting mechanical, optical, and electronic properties across different scales. Developing structure–property processing relationships using machine learning and high-throughput experiments will be essential for a more rational approach to materials design, moving away from simple trial-and-error methods.

### 7.1 Enzymatic engineering for green processing

Future developments in the enzymatic degradation of these biopolymers hinge on refining oxidative enzymes. Engineering high-performance variants of laccases, peroxidases, and tyrosinases with enhanced stability and catalytic efficiency under industrial conditions will enable selective, eco-friendly breakdown of biopolymer matrices.^109^ Enzyme immobilization and nanozyme-based hybrid systems are expected to play a key role in industrial bio-catalysis, offering both cost-effectiveness and reusability.^111^

Exploration into fungal and mycelium-assisted enzymatic systems could provide scalable options for decomposing complex biomaterials.^280^

A critical research gap is the limited selectivity of current oxidative enzymes, which often produce uncontrolled crosslinking or degradation. Future work must prioritize engineering enzymes with programmable specificity to enable precise depolymerization or functionalization pathways. Additionally, industrial scalability remains constrained by enzyme cost, stability in harsh environments, and challenges in continuous-flow bioprocessing.

### 7.2 Bioinspired polymer applications in advanced materials

PDA's surface-functionalization properties are increasingly integrated into high-performance coatings, biomedical devices, and smart composites. Future applications will benefit from improved control over polymerization kinetics and surface morphology to ensure functional performance across varied substrates.^250^ PDA will likely play a prominent role in next-generation soft robotics and bioelectronics.^299^

Lignin and tannin derivatives emerging as eco-efficient adhesives and resins in construction and automotive industries, demanding optimization of their mechanical and rheological properties.^75^ Lignin-based materials could also serve in energy devices, such as dielectric layers in organic field-effect transistors.^300^ Melanin-inspired polymers are promising in electrochemical applications, like biosensing and bio-batteries.^174^

However, a persistent challenge is maintaining consistent batch-to-batch performance because of natural variability in biomass feedstocks. Future approaches should involve standardizing feedstocks, genetically engineering biomass sources, and hybridizing with synthetic monomers to enable tunable and predictable outcomes performance.

### 7.3 Green chemistry and solvent systems

Deep eutectic solvents (DES) and ionic liquids offer promising green alternatives for extracting and processing bioinspired polymers. Their role in sustainable materials synthesis is growing, especially in producing smart polymers with tunable phase transition behaviors.^301^ Integration with continuous-flow systems enhances process efficiency and reduces solvent waste.^86^

Green solvent-compatible photopolymerization and electrospinning techniques are also emerging as key methods for fabricating advanced bio-composites for healthcare and environmental use.^302^ Despite these advances, the toxicity, recyclability, and long-term environmental fate of many DES and ionic liquids remain insufficiently characterized. Future research must prioritize solvent recovery systems, toxicity benchmarking, and standardized environmental impact assessments to ensure that “green” solvents do not introduce new ecological burdens.

### 7.4 Circular economy and smart biopolymers

Developing bio-based materials with engineered end-of-life recyclability is central to circular economic goals. Smart biopolymer systems with self-healing, shape-memory, or environmental sensing capabilities will become prevalent in consumer and industrial products.^303^

Examples include biodegradable packaging with integrated colorimetric sensors for food freshness,^304^ or banana fiber-reinforced composites for agricultural use.^305^ Future products will also involve bio-based 3D printing filaments for additive manufacturing in electronics and architecture. The main challenge is creating materials that balance durability during use with degradability at end of life. This balance requires the use of dynamic covalent chemistries, reversible crosslinking networks, and stimuli-responsive degradation triggers that can be activated on command demand.

### 7.5 Interdisciplinary material innovation

Next-generation materials will stem from interdisciplinary collaboration involving biotechnologists, chemists, engineers, and data scientists. Dynamic bonding polymers, capable of adapting to stress or self-healing, will redefine the boundaries of functional materials.^306^

Machine learning-driven material screening, sustainable polymer platform design, and circular economy modeling for polymer lifecycles will likely define the roadmap for sustainable innovation. Applications include bioelectronic interfaces, renewable energy storage systems, and adaptive biomedical implants. The main bottleneck is the lack of integrated datasets linking molecular structure, processing conditions, and long-term performance metrics. Developing open-access databases and datasets suitable for AI will accelerate discovery and shorten development cycles. Additionally, regulatory frameworks for bio-based materials lag behind technological advancements, creating uncertainty about industry adoption. Establishing clearer standards for biodegradability, toxicity, and lifecycle metrics will be crucial to advancing commercialization.^307,308^

Table 2 summarizes key research gaps across major thematic areas and highlights prioritized strategies aimed at advancing structural control, enzymatic processing, material design, green solvents, circularity, and data standards to accelerate progress in sustainable polymer science.

**Table 2:** Critical bottlenecks, research gaps, and priority strategies for bioinspired polymers

Category	Key bottlenecks/research gaps	Priority strategies	Expected impact
Structural control & characterization	– Heterogeneous polymer structures	– Develop multimodal spectroscopy + ML-assisted structural mapping	Reliable material performance; regulatory readiness; improved design rules
	– Poor reproducibility across biomass sources	– Standardize biomass feedstocks	
	– Limited multi-scale characterization tools	– Create open-access structural databases	
Enzymatic processing & degradation	– Low selectivity of oxidative enzymes	– Engineer programmable laccases/peroxidases	Scalable green processing; controlled depolymerization; reduced waste
	– Instability under industrial conditions	– Develop enzyme–nanozyme cascades	
	– Limited continuous-flow compatibility	– Immobilization for reusability	
Material design & functionalization	– Lack of predictive structure–property relationships	– High-throughput screening + computational modeling	Tailored materials for electronics, coatings, energy devices
	– Empirical trial-and-error dominates	– Hybrid bio-synthetic polymer systems	
	– Variability in mechanical/electronic properties	– Tunable crosslinking chemistries	
Green solvent systems	– Incomplete toxicity and biodegradability data	– Develop bio-derived DES	Safer processing; reduced ecological footprint; industrial viability
	– Limited solvent recovery technologies	– Implement solvent recycling loops	
	– High cost of ionic liquids	– Standardize environmental impact metrics	
Circularity & end-of-life design	– Difficulty balancing durability *vs.* degradability	– Stimuli-responsive degradation triggers	True circular economy materials; reduced landfill burden
	– Lack of reversible chemistries	– Dynamic covalent networks	
	– Limited recycling pathways	– Enzymatic recycling platforms	
Data, modeling & regulation	– Fragmented datasets	– Build AI-ready datasets	Faster innovation cycles; improved safety; smoother commercialization
	– No unified standards for biodegradability or toxicity	– Develop lifecycle modeling tools	
	– Slow regulatory adaptation	– Establish global standards for bio-based materials	

## 8 Conclusions

This review examined the structural properties, applications, and enzymatic breakdown of polydopamine, lignins, tannins, and melanins, highlighting their multifaceted roles in materials science, environmental sustainability, and bioengineering. Although enzymatic methods using laccases, peroxidases, and lignin-degrading enzymes show strong potential, differences in degradation rates and structural complexity still limit efficient biodegradation and recycling. Tannins degrade the fastest due to their simple hydrolyzable structures, PDA exhibits moderate degradability suitable for coatings and biomedical uses, lignins require specialized oxidative systems for partial depolymerization, and melanins remain the most resistant, offering long-term stability for protective and electronic applications. By comparing these polymers systematically, this review outlines their strengths and limitations and provides a foundation for enzyme-guided recycling strategies and the development of sustainable, bio-based materials. This aligns with green chemistry principles by promoting environmentally responsible alternatives to synthetic plastics and supporting circular material flows.

Looking forward, advancing bioinspired polymers will require addressing several persistent challenges that currently limit their broader adoption. Structural heterogeneity, limited enzymatic selectivity, and the absence of predictive structure–property relationships remain major obstacles for both material design and end-of-life processing. Developing standardized feedstocks, improving multi-scale characterization, and engineering more selective oxidative enzymes will be essential for improving reproducibility and enabling controlled degradation.

Equally important is the need for scalable and environmentally responsible processing technologies. Progress in deep eutectic solvents, continuous-flow systems, and hybrid enzyme–nanozyme platforms offers promising routes toward greener manufacturing. Designing reversible crosslinking chemistries and stimuli-responsive degradation pathways will also be key to achieving true circularity in bio-based materials.

As interdisciplinary approaches continue to converge, combining biotechnology, materials science, computational modeling, and sustainability analytics, bioinspired polymers are poised to move from niche alternatives to core components of next-generation sustainable technologies. Integrating enzyme engineering, predictive modeling, and circular-economy principles will enable these natural polymers to transition from laboratory concepts to scalable, high-performance materials for applications in energy, healthcare, electronics, and beyond.

## Author contributions

Ruchika Atri – conceptualization, original draft writing, review and editing. Sascha Putzke – visualization. Dr rer. nat. Frank Simon – conceptualization, visualization, validation, supervision, review, and editing. Dr rer. nat. Cordelia Zimmerer – conceptualization, visualization, validation, supervision, review, and editing.

## Conflicts of interest

There are no conflicts to declare.

## Data Availability

No primary research results, software, or code have been included, and no new data were generated or analyzed as part of this review.
